# *Mycobacterium tuberculosis* Complex Infections in Animals: A Comprehensive Review of Species Distribution and Laboratory Diagnostic Methods

**DOI:** 10.3390/pathogens14101004

**Published:** 2025-10-04

**Authors:** Ewelina Szacawa, Łukasz Radulski, Marcin Weiner, Krzysztof Szulowski, Monika Krajewska-Wędzina

**Affiliations:** 1 Department of Bacteriology and Bacterial Animal Diseases, National Veterinary Research Institute, Partyzantów 57, 24-100 Pulawy, Poland; kszjanow@piwet.pulawy.pl; 2 County Veterinary Inspectorate, C.K. Norwida 17, 24-100 Pulawy, Poland; lukrad91@wp.pl; 3 Department of Technical Sciences, Pope John Paul II State School of Higher Education, 21-500 Biala Podlaska, Poland; m.weiner@dyd.akademiabialska.pl; 4 Department of Food Hygiene of Animal Origin, Faculty of Veterinary Medicine, University of Life Sciences, Akademicka 12, 20-033 Lublin, Poland; monika.krajewska@up.lublin.pl

**Keywords:** *Mycobacterium tuberculosis* complex, MTBC, bovine tuberculosis, animal diagnostics, molecular typing, zoonoses

## Abstract

The *Mycobacterium tuberculosis* complex (MTBC) represents one of the most significant bacterial pathogen groups affecting both animals and humans worldwide. This review provides a comprehensive analysis of MTBC species distribution across different animal hosts and evaluates current laboratory diagnostic methodologies for pathogen detection and identification. The complex comprises seven primary species: *Mycobacterium bovis*, *M. caprae*, *M. tuberculosis*, *M. microti*, *M. canettii*, *M. africanum*, and *M. pinnipedii*, each exhibiting distinct host preferences, geographical distributions, and pathogenic characteristics. Despite sharing >99% genetic homology, these species demonstrate variable biochemical properties, morphological features, and pathogenicity profiles across mammalian species. Current diagnostic approaches encompass both traditional culture-based methods and advanced molecular techniques, including whole genome sequencing. This review emphasises the critical importance of rapid, accurate detection methods for effective tuberculosis surveillance and control programmes in veterinary and public health contexts.

## 1. Introduction

Rod-shaped, acid-fast bacteria of the genus *Mycobacterium*, belonging to the class *Actinomycetes*, containing mycolic acids, constitute some of the most formidable pathogens in both veterinary and human medicine. With over 140 recognised species, this genus encompasses organisms with highly variable virulence characteristics that depend on individual strain properties and host susceptibility factors. The most virulent mycobacteria belong to the *Mycobacterium tuberculosis* complex (MTBC). Although they differ phenotypically, they are grouped in the complex according to genetic similarity. MTBC bacteria serve as the causative agent of tuberculosis (TB) in humans and animals including livestock, wild and captive animals, and even primates [1].

MTBC comprises seven principal species: *Mycobacterium bovis*, *M. caprae*, *M. tuberculosis*, *M. microti*, *M. canettii*, *M. africanum*, and *M. pinnipedii* [2]. These bacterial species are characterised by remarkable genetic homology (>99% nucleotide similarity) yet exhibit significant variation in their biochemical profiles, morphological characteristics, resistance to chemotherapeutic agents, and pathogenic potential across different mammalian hosts.

Bovine tuberculosis (bTB) is one of the most significant bacterial infectious diseases. It is widespread almost all over the world, with the highest prevalence in Africa and Asia, and the lowest in Europe, where many countries have achieved disease-free status. bTB causes economic losses all over the world. These are mainly generated by eradication programmes, transport restrictions, and culling infected animals in developed countries. Generally, the exact costs are not calculated, but recently, the EFSA reported sample costs incurred by selected countries. The costs of eradication, i.e., culling and compensation in 2007–2011 in Italy, were estimated at above 6 million EUR [3]. In Great Britain, the costs of controlling the disease in 2010–2011 were more than 100 million GBP, and Ireland bears costs of 80 million GBP each year. Moreover, the costs of surveillance and monitoring in the UK in the same period of time were 64 million GBP [4]. The disease still causes losses in livestock production, increased mortality, and also trade restrictions in lower-income states [5].

## 2. Species Characteristics and Host Range

### 2.1. Mycobacterium bovis and Mycobacterium caprae

*M. bovis* and *M. caprae* grow dysgonically on solid media, forming dry, rough colonies. These species represent the primary causative agents of bTB, which rank among the most significant bacterial infectious diseases affecting free-living and captive animals alongside Johne’s disease, yersiniosis, leptospirosis, brucellosis, pasteurellosis, anthrax, salmonellosis, and colibacillosis [6,7]. *M. bovis* acts as the main causative agent of bTB in cattle, and to a lesser extent, it is also caused by *M. caprae*. *M. bovis* has also been isolated from several other domestic and wildlife species including sheep, goats, dogs, cats, camelids, horses, bison, wild boars, red deer, fallow deer, badgers, brushtail possums, and others [8,9,10,11,12,13,14,15]. A decade ago, *Mycobacterium caprae* was only recorded on the European continent [16]. The infection mainly occurs via the respiratory route from close contact between animals, and less often via the oral route. The frequency of transmission depends on different biological, behavioural, environmental, or genetic factors [17,18]. Research on bTB in wild boars caused by *M. caprae* has shown a greater number of granulomas in the advanced stage of the disease and a higher number of multinucleated giant cells and acid-fast mycobacteria than in bTB caused by *M. bovis* [19,20].

### 2.2. Mycobacterium tuberculosis

*M. tuberculosis* forms eugonic, dry, and rough colonies on solid media. It is primarily a human pathogen, its infections in animals are relatively uncommon but have been reported in various mammalian species, including dogs, cattle, swine, elephants, and other companion and captive animals [21,22,23,24,25]. In 2022, 4314 cases of TB were recorded in humans in Poland, an upward trend compared to 2021, when there were over 3700 cases; hence, there was a 17.5% increase in cases [26].

### 2.3. Mycobacterium africanum

*M. africanum* demonstrates a highly restricted geographical distribution, being predominantly endemic to West Africa with sporadic occurrences in other regions. This species exhibits biochemical and phenotypic characteristics intermediate between *M. tuberculosis* and *M. bovis*, making differentiation through conventional biochemical methods particularly challenging [27]. Accurate species identification requires molecular methods capable of distinguishing between different lineages. On solid media, it forms dysgonic colonies [28]. Beyond human infections, *M. africanum* has been documented to naturally infect non-human primates and cattle [29].

### 2.4. Mycobacterium microti

Small rodents serve as a natural reservoir for *M. microti*, though this species demonstrates relatively low virulence compared to other MTBC members. Despite its reduced pathogenic potential, *M. microti* can opportunistically infect humans (particularly immunocompromised individuals), domestic animals (cats, dogs, swine, cattle), and various wildlife species, including llamas, ferrets, and badgers. Under experimental conditions, granulomatous lesions in the liver, spleen, lungs, lymph nodes, muscles, and salivary gland were observed [30]. *M. microti* forms slow-growing colonies with a rough morphology. The slow growth characteristics of this organism present significant diagnostic challenges when using conventional culture methods [31,32,33].

### 2.5. Mycobacterium canettii

*M. canettii* forms eugonic, smooth, white, glossy colonies on solid media. It is also known as smooth *M. tuberculosis* (smTB), and exhibits distinctive biochemical and phenotypic characteristics along with higher genetic polymorphism compared to other MTBC species. While proven to cause human infections, there is currently no documented evidence of natural disease induction in animal species [34,35].

### 2.6. Mycobacterium pinnipedii

This species demonstrates a primary host range encompassing marine mammals, particularly pinnipeds (seals, sea lions). It is pathogenic and cases of mortality have been reported. However, zoonotic transmission to humans has been documented. Colony morphology of *M. pinnipedii* on solid media is dysgonic, rough, and flat. Similarly to *M. canettii*, *M. pinnipedii* presents identification challenges when using traditional biochemical differentiation methods [36,37]. An insight into species comprising MTBC, regarding primary host, geographical distribution of the species, its pathogenicity, and main clinical features, is listed in Table 1.

## 3. Epidemiological Significance and Public Health Impact

### 3.1. Geographical Distribution and Socio-Economic Costs

According to recent EFSA surveillance data from 2022, the overall prevalence of MTBC-infected cattle herds across the European Union remains relatively low at 0.61%. However, significant variation exists among member states, with Ireland reporting the highest prevalence (4.6%), followed by Spain (2.5%), while Malta and Cyprus reported no positive herds. Seventeen EU member countries maintain disease-free status, three have established disease-free zones, and seven other countries, along with the United Kingdom (Northern Ireland), lack MTBC-free zones or a status for bTB [38]. In the above-mentioned developed countries, the surveillance data such as herd and/or animal prevalence are reported, but in developing countries, some data can only be obtained on the basis of cross-sectional studies. Such an analysis was made by Ramos et al. It revealed that in North America, there are 33.6% of estimated bTB cases, in Asia, 13.8%, and in Africa, 10.3%. It should be emphasised that there are different cattle densities in these continents, with the highest density in Asia, lower densities in East Africa, Northern Europe, North America, and the lowest density in South America. Different surveillance strategies and diagnostic methods also influenced the analysis, and only a general overview of the disease spread could be obtained [39]. The socio-economic costs of bTB require the estimation of different components. Costs in developed countries, with low bTB prevalence, are mainly related to limitations in animal production and costs of eradication programmes, i.e., costs of skin testing performed by veterinarians [3,40]. On the other hand, in developing countries, for example, Ethiopia, where the prevalence of bTB is high, costs are generated by lower animal production and livestock losses. Moreover, zoonotic TB is highly prevalent in developing countries, further contributing to the cost of treatment [41].

### 3.2. Transmission Dynamics

bTB spreads primarily via the aerogenic route among domestic cattle, though the disease can affect various companion and free-ranging animal species that share grazing areas with infected animals [42]. Disease transmission probability between wildlife and domestic animals depends on multiple factors including species-specific behavioural patterns, herd management practices, and pathogen virulence characteristics. Environmental persistence represents a critical factor in disease transmission, as mycobacteria can survive in soil and bedding materials for several years [43]. This environmental stability contributes significantly to the cross-contamination potential between free-living and domestic animal populations. *M. bovis* infections spread primarily via the aerogenic route, however, the oral route is also possible. Calves can be infected per os from colostrum or milk from an affected mother. Sheep, alpacas, goats, and free-ranging animals, i.e., European bison and wild boar, can also be easily infected with bovine mycobacteria. Infection of sheep and goats usually occurs by the aerogenic route, during direct contact with infected animals. Natural infection in the herd usually occurs as a result of constant, repeated contact with the infectious agent. The source of infection is usually diseased individuals, contaminating the environment with secretions and excretions. Dogs, cats, pigs, and wild boars are usually infected via the oral route through contact with the excretions and secretions of the diseased individuals [19,20].

### 3.3. Zoonotic Potential

The WHO classifies bTB as a direct zoonosis, indicating direct pathogen transmission from infected animals to susceptible vertebrates without intermediate host involvement [44]. Human infection most commonly occurs through direct contact with diseased animals or consumption of contaminated animal products. Notably, infected humans can serve as sources of infection for cattle and other animals, as documented in cases where TB-positive farm workers transmitted the disease to their livestock [36]. Animal and human TB caused by *M. bovis* and *M. caprae* have similar pathological macroscopic changes, and their localisation is similar [19,20].

### 3.4. Evidence of Animal-to-Human and Human-to-Animal Transmission

Zoonoses, or zoonotic diseases, are a broad group of diseases that can be transmitted between animals and humans. These include bacterial, viral, and parasitic zoonoses. Information on zoonoses is regularly updated and published in international and national veterinary journals [45,46,47]. According to the WHO definition, there are three classes of zoonoses: endemic; epidemic, which are sporadic in terms of temporal and spatial distribution; and emerging and re-emerging zoonoses, which existed previously but are rapidly increasing in frequency or geographical range [48]. It is estimated that microbes are responsible for 14 million deaths worldwide each year. In addition to these newer pathogens, “old” ones also continue to threaten us. It also appears that long-standing, seemingly under-control diseases like TB and malaria are making a comeback [49,50].

bTB is an example of a zoonosis, but also anthropozoonosis, where infection occurs from an infected human to an animal. MTBC infections have plagued humanity since ancient times. Long before the appearance of hominids, the disease was present in reptiles and later in fossil mammals. Skeletons from the Mesozoic period bear traces of TB infection [51,52,53].

The reservoir of the bovine bacilli *M. bovis* and *M. caprae* is undoubtedly cattle. Given that these are farm animals with which humans live in close contact, both directly and indirectly, the largest proportion of human TB cases, besides *M. tuberculosis*, is caused by the bovine bacilli. bTB in humans has been reported even in developed countries. In the articles by Kozińska et al., the source of transmission could not be determined. In one publication, the authors described the occurrence of *M. bovis* TB in three patients, and in the other, *M. caprae* TB in one person [54,55]. Krajewska et al. described a severe case of *M. bovis* TB that affected a young farmer who acquired the infection from diseased cattle or at a slaughterhouse where he worked. The pulmonary location of TB in the farmer mentioned above suggests that he acquired the infection through the respiratory route [56]. Treatment during hospitalisation was successful in all of these patients.

bTB is a typical example of zoonosis, an infectious disease that can be transmitted from animals to humans. Shrikrishana et al. described a case of *M. bovis* TB in a woman bitten by a badger, which are widely considered the reservoir of *M. bovis* in the UK. Four months later, her dog developed respiratory symptoms, and the bacilli strain with the same molecular pattern as that from the owner was isolated from the dog’s tracheal lavage [57,58]. An example of another anthropozoonosis-TB, is the occurrence of bTB in two British people who had contact with cats suffering from *M. bovis* TB. The epidemiological investigation did not establish the source of infection for the cats, but it was suspected that infected wild animals, such as badgers or rodents, could have been the source. In Great Britain, badgers are responsible for the spread of bTB among cattle and other species, such as alpacas. In the specific British environment, badgers live near pastures, where they dig earthen burrows, contaminating the surrounding environment. This way, the animals ingest live bacteria through their food, causing infection [59,60].

Bovine bacilli can cause TB in other livestock species. They are highly virulent in nature in goats [61], pigs [62,63], sheep [64], and cats [65,66]. The rarest TB caused by bovine mycobacteria occurs in horses and dogs, although such cases have also been confirmed [67,68].

Cattle have low susceptibility to *M. tuberculosis*, but cases of its infection in cattle have been reported in Poland. A young woman from the border region with Ukraine infected a calf with *M. tuberculosis*. She came daily to milk its mother and had close contact with the calf whenever she visited the barn [69].

### 3.5. Human Populations at Increased Risk and Other Factors

*M. bovis* TB is a zoonosis, and this bovine tuberculi is a zoonotic pathogen subject to mandatory annual monitoring, i.e., List A of Directive 2003/99/EC. The number of bTB cases in humans is underestimated. The incidence of *M. caprae* TB is significantly lower, but this is due to the fact that this mycobacterium is less common in cattle compared to *M. bovis*. The disease most often affects people with immunosuppression, people who have direct contact with animals, or those who consume animal products contaminated with *M. bovis* [70,71]. Weakening or suppressing the activity of the body’s immune system significantly increases the risk of developing TB, especially in HIV-positive individuals. In these patients, the number of CD4^+^ lymphocytes responsible for the cellular immune response decreases significantly [72]. Immunosuppression can activate latent TB infections or accelerate the progression of latent disease into active disease. Gibson et al. described a case of *M. bovis* TB in a pregnant diabetic patient. In this case, the patient’s brother was the vector of infection, but it is nevertheless another example of the increased risk of disease in individuals with weakened immune systems. Diabetes disrupts the body’s defence mechanisms. Abnormal glucose metabolism is associated with impaired function of leukocytes, which are responsible for phagocytosis. Insulin deficiency in diabetics affects glycolysis and consequently, phagocytosis. Phagocytes therefore have a reduced ability to neutralise pathogens, including *M. bovis* [73].

Susceptibility to *M. bovis* infection in both humans and animals is greater in developing countries where bTB control and eradication programmes are lacking. The two main factors contributing to infection are contact with diseased animals and/or consumption of unpasteurised or locally produced products [17,74]. In many developing countries, milk pasteurisation is poorly implemented. In Mexico, almost 30% of milk is sold unpasteurised. This milk is used to produce local delicacies such as artisanal cheeses [72]. More than two decades ago, published data indicated that in San Diego, which is located near the Mexican border, that the number of people infected with *M. bovis* was higher than the average for the United States due to the consumption of dairy products made from unpasteurised milk by the city’s Hispanic population [75].

There is a significant correlation between the location of TB in humans and the path the pathogen uses to enter the body. In cases of direct contact with a sick animal or after handling their carcasses and inoculation of the droplet nuclei with the pathogen, lesions in humans are located in the lungs. Ingestion of raw dairy products from infected cattle is more likely associated with the development of extrapulmonary localisation [17].

Each year, the European Food Safety Authority (EFSA) and the European Centre for Disease Prevention and Control (ECDC) collect information on zoonoses reported in humans in Europe. The report covers zoonoses such as campylobacteriosis, salmonellosis, Shiga toxin-producing *Escherichia coli* infections, yersiniosis, listeriosis, tularemia, echinococcosis, Q fever, West Nile virus infection, brucellosis, *M. bovis* and *M. caprae* TB, trichinellosis, and rabies. Zoonotic TB was reported in 138 patients; most of the cases were caused by *M. bovis*, and fewer by *M. caprae*. Sixty-seven patients were from the European Union, sixty-two infections were acquired outside the EU (with the highest number of confirmed cases in Spain), and nine had an unknown travel status or an unknown country of infection. All patients were hospitalised, and for nineteen, TB proved fatal [38].

It would be a mistake not to mention among the risk factors such professions as veterinarians, people supervising animal breeding, and employees of zoos or animal slaughterhouses [76]. A 32-year-old veterinarian in the UK also developed *M. bovis* TB. He contracted the disease either at his British small animal practice or during a two-week internship in South Africa, where he cared for large and wild animals.

Other risk factors for TB include *M. bovis*, protein malnutrition, alcoholism, drug addiction, and living in poverty.

## 4. Laboratory Diagnostic Methods

### 4.1. Specimen Collection and Initial Processing

In the epidemiology of disease caused by MTBC, an acid-fast mycobacterium, rapid detection and identification of the pathogen is important. The first stage of research is an intradermal screening test. Next, post-mortem examination and culture of pathogenic bacteria are useful to assess disease surveillance. The presence of bacteria belonging to MTBC in animals suspected of having bTB can firstly be determined in clinical samples with the Ziehl–Neelsen staining of microscopic slides or with the use of fluorescence or immunoperoxidase techniques. Next, the presence of TB bacilli can be confirmed by the culture on specific media. Clinical samples should be taken from affected lymph nodes and organs, such as the lungs, liver, or spleen. If any TB-compatible lesions are visible, specific lymph nodes from the head, respiratory, and/or digestive locations should be taken for the culture. Once specific colonies suggestive of MTBC are obtained, species identification of isolates should be performed [77].

### 4.2. Culture Methods

#### 4.2.1. Solid Media Culture

Culture remains the “gold standard” for bTB diagnosis, with solid media being most commonly employed. Egg-based media containing pyruvate or both pyruvate and glycerol (Stonebrink, Löwenstein-Jensen) represent standard options, while solid agar plates such as Middlebrook media provide alternative culture systems. Mycobacterial growth typically occurs within 3–6 weeks, necessitating incubation periods of at least 6 weeks at 37 °C. Initial identification relies on characteristic colony morphology and growth patterns, followed by species-specific PCR confirmation, as biochemical identification proves impractical due to slow growth characteristics [78].

#### 4.2.2. Liquid Culture Systems

Liquid culture systems can be utilised in laboratories equipped with specialised instrumentation capable of measuring fluorometric signals from specific liquid media during mycobacterial growth. In the MGIT system (Becton Dickinson, Franklin Lakes, NJ, USA), decontamination of samples is achieved with the use of MycoPrep containing N-acetylo–L-cysteine and 2% NaOH, and culture is carried out, for example, in broths provided by the manufacturer or in 7H9 broth. While growth occurs more rapidly in liquid media compared to solid systems, PCR confirmation remains essential since characteristic morphological features cannot be observed in liquid culture systems [78].

### 4.3. Phenotypic Identification Methods

#### 4.3.1. Biochemical Testing

Biochemical tests (biotyping) enable differentiation among MTBC species obtained after culture of sample, and their distinction from non-tuberculous mycobacteria is based on metabolic differences. For example, *M. bovis* is negative in niacin production and nitrate reduction. It is positive for urease and negative for nicotinamidase and pyrazinamidase in the amidase test. However, these methods are impractical for routine use due to time-consuming procedures and lengthy incubation requirements. However, there is another possible way to differentiate MTBC from non-tuberculosis bacteria, through the use of a medium containing p-nitrobenzoic acid, in which MTBC, unlike other bacteria, does not grow [78].

#### 4.3.2. Immunochromatographic Methods

Immunochromatographic assays serve as auxiliary tools for rapid MTBC identification, utilising antibodies tagged with visible markers such as colloidal gold. These antibodies bind to specific antigens in test samples, forming complexes that migrate along test strips and produce visible colour reactions upon capture by fixed antibodies [79,80]. Regarding *M. bovis*, the targeted protein is MPB64, and for *M. tuberculosis*, it is MPT64 [81,82].

#### 4.3.3. Chromatographic Analysis

High-performance liquid chromatography (HPLC) enables qualitative and quantitative analysis of mycolic acids, which are integral cell wall components of *Mycobacterium* species. With this technique it is possible to separate the patterns specific to *M. bovis* and *M. tuberculosis*. It allows for species and subspecies identification within minutes. However, equipment and reagent costs are substantial, and result interpretation can be challenging [83,84].

#### 4.3.4. Matrix-Assisted Laser Desorption/Ionisation Time-of-Flight Mass Spectrometry

MALDI-TOF MS analyses bacterial protein profiles and compares them against reference spectra in established databases. While this method can identify MTBC at the complex level, species-level differentiation within the complex remains limited. Most non-tuberculous mycobacteria can be distinguished at the species level, with some exceptions. Protocols enable the detection in solid and liquid media [85,86]. Bruker Daltonics and VITEK MS databases for described in vitro diagnostics are mostly used. The databases are able to correctly identify MTBC members, although some differences in accuracy values can be seen. Observed disagreements are caused mostly by different protein extraction protocols [80,87,88].

### 4.4. Molecular Genetic Methods

#### 4.4.1. Polymerase Chain Reaction

PCR-based techniques enable direct detection of MTBC genetic material in tissue samples, providing rapid confirmation of pathogen presence. These methods offer valuable epidemiological information that supports bTB control efforts by identifying potential links between diseased animals, detecting outbreak patterns, and identifying laboratory cross-contamination events. The real-time PCR is widely used, mostly based on IS*6110* and IS*1081*, repetitive insertion sequences, and the *mpb64* gene, which ensures high sensitivity of the reaction. The *mpb64* is often used with the previously mentioned sequences to enhance the detection rates [89,90]. Moreover, to obtain the most accurate results, the proper DNA-extraction process is significant; and it should employ a mechanical or chemical lysis step [91].

#### 4.4.2. Spoligotyping

Spoligotyping is a PCR-based method that assesses polymorphism within the Direct Repeat (DR) chromosomal region of MTBC. Results are compared against international databases such as the *M. bovis* spoligotype database for strain characterisation and tracking the source of transmission of MTBC members of animal origin or SITVIT2 regarding *M. tuberculosis* [92,93,94,95,96]. Examining the genetic variations between strains from various outbreaks using the described method can play a key role in understanding how the infection spreads and may also be essential for enhancing strategies to eliminate bTB in livestock.

#### 4.4.3. Mycobacterial Interspersed Repetitive Units-Variable Number of Tandem Repeats Analysis

MIRU-VNTR analysis examines specific genomic loci containing repeated motifs with variable copy numbers among strains of the same species. *M. bovis*/*M. caprae* strains are typed by VNTR-MIRU with ETRA-E, and seven additional VNTR/MIRU markers should be added to obtain more accurate genetic profiles [97,98]. Due to limitations in discriminatory power, for example, in large cross-sectional population studies and species differentiation when used alone, MIRU-VNTR analysis is often combined with other typing methods, such as spoligotyping, for enhanced strain differentiation [99].

#### 4.4.4. Whole-Genome Sequencing

Whole-Genome Sequencing (WGS) is a high-resolution alternative to traditional molecular methods [100]. Due to the high genomic similarity of the mycobacterial species of MTBC, WGS is suitable for precise epidemiological investigation/links. The possibility of using the whole genome for analysis instead of information received from previous molecular techniques enables a much higher resolution, especially using Next-Generation Sequencing (NGS) [100,101]. The overview of the laboratory diagnostic methods for MTBC detection and identification including its characteristics and the time of analysis is presented in Table 2.

## 5. Diagnostic Challenges and Future Perspectives

The diagnostics of bTB are challenging, starting from limitations of current field-deployable tests concerning moderate sensitivity and the specificity of tuberculin skin tests. Differentiation among MTBC species using biochemical tests is generally not conducted due to its time-consuming nature. Moreover, the genetic conservation within MTBC species (>99% similarity) presents inherent limitations for molecular differentiation methods. However, various DNA fingerprinting approaches offer different discriminatory potentials and serve distinct epidemiological purposes. Advances in Next-Generation Sequencing technologies continue to enhance our ability to perform precise epidemiological investigations and strain tracking. bTB is a transboundary disease, if standardised protocols across countries were introduced, it would certainly help in consistent data reporting, efficient livestock trade, and control in wildlife movement. Nevertheless, we should remember that standardisation should be adapted to the needs of particular countries. Nowadays, point-of-care diagnostics generally seem promising and would accelerate on-site bTB diagnosis, but it is still in development, and there are no available methods with sufficient sensitivity and specificity for bTB. The most promising method would be the integration of genomics into surveillance systems. It could enhance outbreak investigations and enable tracing of the specific outbreaks. These developments promise improved outbreak investigation capabilities, more effective bTB control strategies, and improved disease modelling in both veterinary and public health contexts.

## 6. Conclusions

MTBC represents a diverse group of pathogens with significant implications for animal and human health. Despite remarkable genetic similarity, individual species demonstrate distinct host preferences, geographical distributions, and pathogenic characteristics. Effective bTB surveillance and control programmes require integration of traditional culture methods with advanced molecular diagnostic techniques. Current diagnostic approaches encompass a spectrum from conventional culture and biochemical methods to sophisticated molecular typing and Whole-Genome Sequencing. The selection of appropriate diagnostic strategies depends on specific epidemiological objectives and available resources. Continued advancement in molecular diagnostic technologies, particularly Whole-Genome Sequencing, promises enhanced precision in epidemiological investigations. However, the fundamental importance of culture methods for definitive diagnosis ensures their continued relevance in comprehensive diagnostic approaches.

## Figures and Tables

**Table 1:** Overview of primary species belonging to the *Mycobacterium tuberculosis* complex.

Primary Hosts	Main Geographical Distribution	Pathogenicity	Key Clinical Features
*Mycobacterium bovis*			
Cattle; Wildlife animals (e.g., deer, boar, badgers); Domestic animals (e.g., dogs, cats);	Global	Mostly pathogenic to cattle; Zoonotic potential	Causes bTB; Respiratory transmission; Forms granulomas; Zoonotic transmission possible; Dry rough colonies
*Mycobacterium caprae*			
Goats; Cattle; Wild boar; Red deer; Other wildlife;	Mainly Europe	Pathogenic to animals	Causes bTB (less commonly than *M. bovis*); Forms granulomas;
*Mycobacterium tuberculosis*			
Humans (primary); Animals (e.g., dogs, elephants, cattle);	Global	High pathogenicity to humans; Rare pathogenicity to animals;	Causes human TB; Respiratory Transmission; Zoonosis;
*Mycobacterium africanum*			
Humans (primary); Non-human primates; Cattle;	West Africa (endemic), sporadic elsewhere	Moderate pathogenicity;	Restricted to certain regions; Zoonosis;
*Mycobacterium microti*			
Rodents (primary); Cats; Dogs; Swine;	Reported in Europe	Low virulence;	Granulomatous lesions in the liver, spleen, lungs, lymph nodes, muscles, and salivary gland under experimental conditions;
*Mycobacterium canettii*			
Humans (only known host);	East Africa (mainly)	Pathogenic	Known as “smooth TB”;
*Mycobacterium pinnipedii*			
Marine mammals (e.g., seals, sea lions)	Coastal regions (especially the Southern Hemisphere)	Pathogenic	Zoonosis

**Table 2:** Laboratory diagnostic methods for MTBC detection and identification.

Method	Time of Analysis	Characteristics
Culture methods		
Solid media culture	3–6 weeks	High specificity; Acts as the gold standard; Characteristic morphology visible; Slow growth; Long incubation required.
Liquid culture system	Less than solid media	More rapid growth detection than solid media; No morphological features; Requires specialised equipment; PCR confirmation needed.
Phenotypic identification		
Biochemical tests	Days/weeks	Cost-effective; Species differentiation possible; Limited discrimination; Time-consuming; Long incubation.
Immunochromatographic assays	Minutes/hours	Rapid detection; Quick screening; Field testing; Act as an auxiliary tool only.
HPLC analysis	Minutes	Very quick; Species and subspecies identification; Expensive equipment; Requires interpretation by a specialist.
MALDI-TOF MS	Minutes	Suitable for solid and liquid media; Proper for non-tuberculous mycobacteria; Limited species-level differentiation within MTBC.
Molecular methods		
PCR	Hours	Rapid diagnosis; Sensitive; Analysis direct from tissue; Contamination detection; Requires specialised equipment; Requires specialised result analysis.
Spoligotyping	1–2 days	Suitable for strain characterisation; International database comparison; Epidemiological tracking; Limited discriminatory power when used alone.
MIRU-VNTR Analysis	1–2 days	Good strain differentiation when combined with other methods; Limited discriminatory power when used alone.
Whole-Genome Sequencing	Days	Highest resolution; Precise epidemiological investigation; Precise strain tracking; Expensive; Complicated; Requires specialised equipment and staff.

## Data Availability

No new data were created or analysed in this study. Data sharing is not applicable to this article.

## Abbreviations

The following abbreviations are used in this manuscript:

MTBC	*Mycobacterium tuberculosis* complex
EFSA	European Food Safety Authority
WHO	World Health Organization
bTB	bovine tuberculosis
HPLC	High-performance liquid chromatography
MALDI-TOF MS	Matrix-Assisted Laser Desorption/Ionisation Time-of-Flight Mass Spectrometry
PCR	Polymerase Chain Reaction
MIRU-VNTR	Mycobacterial Interspersed Repetitive Units-Variable Number of Tandem Repeats
WGS	Whole-Genome Sequencing
